# Serum copper, zinc and selenium and their ratios as predictors of pneumonia death risk in men: the Kuopio Ischaemic Heart Disease Risk Factor Study

**DOI:** 10.1007/s15010-025-02596-8

**Published:** 2025-07-10

**Authors:** Jaakko T. Laine, Tomi-Pekka Tuomainen, Jukka T. Salonen, Jyrki K. Virtanen

**Affiliations:** 1 Institute of Public Health and Clinical Nutrition, University of Eastern Finland, P.O. Box 1627, Kuopio, 70211 Finland; 2 Faculty of Medicine, Department of Public Health, University of Helsinki, Helsinki, Finland; 3 MAS-Metabolic Analytical Services Oy, Helsinki, Finland

**Keywords:** Copper, Zinc, Selenium, Pneumonia mortality, Population study

## Abstract

**Purpose:**

This study aimed to assess the associations between serum concentrations of copper, zinc and selenium, and pneumonia death risk.

**Methods:**

Study included 2088 men from the Kuopio Ischaemic Heart Disease Risk Factor Study aged 42–60 years. Pneumonia deaths were collected by computer linkage to the national Causes of Death Register. Cox proportional hazards regression models, adjusted for multiple variables, were used for analysis.

**Results:**

During a mean follow-up of 21.7 years (SD 7.5 years), 139 pneumonia deaths occurred. The multivariable-adjusted hazard ratio for pneumonia death in the highest serum copper-to-zinc-ratio and copper concentration tertiles were 1.75 (95% CI: 1.13–2.71) and 1.64 (95% CI: 1.08–2.50), respectively. Serum zinc concentration showed a statistically significant association with pneumonia death, with the lowest risk observed in the second tertile and no further decrease in risk in the highest tertile. Serum copper-to-selenium ratio nor selenium concentrations were associated with pneumonia death risk.

**Conclusions:**

Our findings suggest that a higher serum copper-to-zinc-ratio and higher serum copper concentration are associated with increased risk of pneumonia death, while a higher serum zinc concentration is linked to a decreased risk of pneumonia death in middle-aged and older men.

**Supplementary Information:**

The online version contains supplementary material available at 10.1007/s15010-025-02596-8.

## Introduction

Lower respiratory tract infections are the most common cause of infectious disease mortality worldwide [1]. The number of pneumonia-related hospital deaths among older adults was estimated in 2015 at 1.1 million based on global data on pneumonia hospital admissions [2]. Several factors may contribute to increased susceptibility to pneumonia and pneumonia mortality in the elderly, including co-morbidities with chronic diseases and age-related immune system decline [3].

Copper (Cu), zinc (Zn), and selenium (Se) are essential trace elements known to have a significant impact on immune and antioxidative systems [4, 5]. The combined biomarkers of Cu/Zn-ratio and Cu/Se-ratio have been proposed as more accurate indicators of inflammation, aging, and oxidative stress than serum Cu, Zn, and Se concentrations alone, although results have been inconclusive [6]. Increased Cu/Zn-ratio and serum Cu concentration have been identified as predictive factors for pneumonia [7], and all-cause mortality in the elderly [8]. On the other hand, Se deficiency has been associated with an increased risk of viral infections and suggested as a risk factor for infection disease progression, poor recovery, and increased mortality [9].

However, to our knowledge, the predictive value of serum Cu, Zn, and Se concentrations and their ratios for pneumonia-related mortality in the elderly has not been investigated in a prospective population-based cohort study. Therefore, the objective of our study was to examine the associations between serum concentrations of Cu, Zn, Se, and their ratios (Cu/Zn, Cu/Se) and the risk of pneumonia death in the population-based Kuopio Ischaemic Heart Disease Risk Factor Study (KIHD) cohort. Additionally, we examined the individual associations of serum Cu, Zn, and Se concentrations with the risk of pneumonia death.

## Methods

### Study population

The KIHD was designed to investigate risk factors for cardiovascular disease (CVD), atherosclerosis, and related outcomes in a population-based sample of men from eastern Finland [10]. The baseline examinations were carried out in 1984–1989. A total of 2682 men who were 42, 48, 54, or 60 years old at baseline (83% of those eligible) were recruited in 2 cohorts. The first cohort consisted of 1166 men who were 54 years old and enrolled in 1984–1986, and the second cohort included 1516 men who were 42, 48, 54, or 60 years old and enrolled in 1986–1989. The baseline characteristics of the entire study population were described previously [10].

Data on serum Cu, Zn and Se concentrations were available for 2554 men. Participants who had the serum CRP > 10 mg/L (*n* = 71), indicating an acute inflammation, or those with a diagnosis of chronic diseases that are associated with increased risk of pneumonia, such as chronic bronchitis (*n* = 193), lung tuberculosis (*n* = 88), bronchial asthma (*n* = 60), history of cancer (*n* = 39) or rheumatoid arthritis with inflammatory and antirheumatic therapy (*n* = 15) at entry, were excluded, leaving 2088 men for the analysis (Supplementary Fig. 1). In the sensitivity analyses, we further excluded men with potentially increased risk of pneumonia, including men with history of ischemic heart disease, stroke, liver or pancreas disease or diabetes (*n* = 574), leaving 1514 men for these analyses.

### Data collection

Fasting venous blood samples were drawn between 08.00 and 10.00 at baseline. Subjects were instructed to abstain from ingesting alcohol for 3 days and from smoking and eating for 12 h before providing the sample. Copper-free needles and tubes were used for collecting and storing the blood samples. The subjects rested in a supine position for 30 min before blood sampling. A tourniquet was not used.

Detailed descriptions of the assessment of medical history and medications [11], family history of diseases [11], smoking [11], alcohol consumption [11], serum ferritin [11], and physical activity [12], at baseline have been published. Education and annual income were obtained from a self-administered questionnaire. Height and weight were measured by a study nurse. BMI was computed as the ratio of weight in kilograms to the square of height in meters. Dietary intakes at baseline were assessed with 4-day food records [13]. An immunometric assay (Immulite High Sensitivity C-reactive Protein Assay; DPC, Los Angeles, USA) was used to measure serum high-sensitivity CRP.

### Determination of serum copper, zinc and selenium concentrations

Serum Cu, Zn and Se concentrations were determined by atomic absorption spectrometry from frozen samples stored at − 20 C° for 1–5 years prior to analyses. Serum Zn concentrations were determined in the same batches with Cu, method for which has been described before [14]. The PerkinElmer 306 atomic absorption spectrophotometer (Norwalk, Connecticut, USA) was used for measurements. Control serum samples (Seronorm Nycomed, Oslo, Norway) with known concentrations of the elements were included in all daily batches. The reference standards were dissolved in 5% glycerol. The between-batch coefficient of variation was 4.0%. Se concentration was measured with a Perkin-Elmer 5000 atomic absorption spectrophotometer HGA 500 by the graphite furnace technique with a Zeeman background correction and pyrolytically coated graphite tubes with a platform. The between batch coefficient of variation was 5.1% [14].

### Ascertainment of follow-up events

Pneumonia deaths were ascertained by computer linkage to the national Causes of Death Register using the Finnish personal identification code (social security number). The pneumonia deaths from the study entry to 31 December, 2013 were coded according to the International Classification of Diseases (8th, 9th and 10th revision) codes.

### Statistical analysis

Chi-square tests (for categorical variables) and linear regression (for continuous variables) were used to examine the associations between serum Cu/Zn- and Cu/Se-ratio and baseline characteristics in tertiles of serum Cu/Zn- and Cu/Se-ratio. Correlations between serum Cu, Zn and Se were estimated by Spearman’s correlation coefficients. Multivariable-adjusted Cox proportional hazards regression models were used to analyze the association between baseline serum Cu/Zn- and Cu/Se-ratio (in tertiles) and pneumonia death and further the association between baseline Cu, Zn and Se concentrations (in tertiles) and pneumonia death. Schoenfeld residuals did not indicate significant evidence of violation of the proportional hazards assumption. Participants contributed follow-up time until pneumonia death or the end of follow-up, which ever came first. The potential confounders were selected based on risk factors for pneumonia identified in previous studies [2] and our previous study regarding Cu/Zn-ratio and risk of incident infection [15]. Model 1 included age (years) and baseline examination year. Model 2 included model 1 plus history of coronary heart disease, stroke, diabetes, liver or pancreas disease (yes/no); smoking (never smoker, previous smoker, current smoker < 20 cigarettes/day, current smoker *≥* 20 cigarettes/day); education (years); income (euros/year); intake of alcohol (g/week); leisure-time physical activity (kcal/day); and BMI (kg/m^2^). In the sensitivity analyses, we excluded men with a history of ischemic heart disease, stroke, cancer, diabetes, or liver or pancreas disease (*n* = 574), and adjusted the model 2 further for albumin. Among the participants, 47 men (2.3%) had missing data in smoking, 33 (1.6%) in annual income, 28 (1.3%) in albumin, 10 (0.5%) in leisure-time physical activity, 9 (0.4%) in BMI, 3 (0.1%) in alcohol consumption, and 2 (0.1%) in education. Little’s Missing Completely at Random-test suggested (*p* < 0.001) that missingness was not completely random, so multiple imputation was used to replace the missing values. Statistical analyses were performed using SPSS software version 29 for Windows (Armonk, NY, IBM Corp.). All *P*-values were two-sided.

## Results

The present study included 2088 men with a mean age of 52.8 years (standard deviation (SD) 5.2 years). Their mean serum Cu, Zn and Se concentrations were 17.4 µmol/L (SD 2.7 µmol/L, range 7.2–36.5 µmol/L), 14.4 µmol/L (SD 1.8 µmol/L, range 8.3–22.9 µmol/L) and 1.3 µmol/L (SD 0.3 µmol/L, range 0.5–3.1 µmol/L), respectively. The mean serum Cu/Zn- and Cu/Se-ratios were 1.2 (SD 0.2, range 0.5–3.0) and 11.3 (SD 3.5, range 3.9–31.8), respectively. The correlation coefficients were − 0.05 between serum Cu and Zn concentrations, -0.01 between serum Cu and Se concentrations and 0.05 between serum Zn and Se concentrations. Table 1 shows the baseline characteristics of the participants, providing an overview of their key demographic and lifestyle factors. Supplementary Tables 1 and 2 show the characteristics based on tertiles of serum Cu/Zn- and Cu/Se-ratios. Men with higher Cu/Zn-ratio and Cu/Se-ratio were more likely to be older, be current smokers, be less educated and have a lower income than those with a lower serum Cu/Zn-ratio and Cu/Se-ratio. A higher Cu/Zn-ratio was associated with a higher consumption of alcohol and a higher intake of fish but with a lower intake of fruits, berries, vegetables and roots. Men with a higher Cu/Se-ratio were physically less active and had a lower intake of meat and zinc but a higher intake of dairy products than those with lower Cu/Se-ratio. The correlation coefficient between Zn intake and serum Zn concentration was 0.07. We did not have information of Cu and Se intakes.

**Table 1:** Baseline characteristics of 2088 men from the Kuopio Ischaemic Heart Disease Risk Factor Study

Characteristic	Mean *±* SD or percentages
Sociodemographic and lifestyle factors	
Age, years	52.8 *±* 5.2
Education, years	8.7 *±* 3.5
Income, 1000€/year	13.5 *±* 9.0
BMI, kg/m^2^	26.8 *±* 3.5
Leisure time physical activity kcal/day	141 *±* 170
Multivitamin use, %	1.1
Current smoker, %	29
Alcohol intake, g/week	73 *±* 117
Disease history and serum biomarkers	
History of ischaemic heart disease, stroke, cancer or diabetes, %	28
Serum C-reactive protein, mg/L	1.8 *±* 1.8
Serum albumin, g/L	42 *±* 3.5
Serum Cu, µmol/L	17.4 *±* 2.7
Serum Zn, µmol/L	14.4 *±* 1.8
Serum Se, µmol/L	1.3 *±* 0.3
Serum Cu/Zn	1.2 *±* 0.2
Serum Cu/Se	11.3 *±* 3.5
Dietary intakes	
Energy intake, kcal/day	2436 *±* 613
Meat, g/d^1^	159 *±* 81
Dairy, g/d	709 *±* 356
Fish, g/d	46 *±* 54
Eggs, g/d	33 *±* 26
Grains, g/d	254 *±* 93
Vegetable margarines and oils, g/d	20 *±* 17
Butter, g/d	33 *±* 26
Fruits, berries, vegetables, roots, g/d	418 *±* 179
Zn, mg/d^2^	15 *±* 5

^1^ Includes red meat, white meat, game and offal

^2^ Value adjusted for total energy intake using the residual method

During the mean follow-up time of 21.7 years (SD 7.5 years) a total of 139 pneumonia deaths were diagnosed. After adjustment for age and examination year, in the Cox regression model (Table 2, model 1) there was a statistically significant higher risk of pneumonia death with an increased Cu/Zn-ratio (*P*-trend across tertiles = 0.001). The hazard ratio (HR) in the highest vs. the lowest tertile was 2.06 (95% CI: 1.35, 3.15; (Table 2, model 1). This association was attenuated but remained statistically significant when the model was further adjusted for social and lifestyle factors and for several comorbidities at baseline. The multivariate-adjusted HR in the highest vs. lowest tertile of Cu/Zn-ratio was 1.76 (95% CI: 1.14, 2.72; *P*-trend = 0.010) (Table 2, model 2). The association was not altered when serum albumin, an indicator of poor nutritional status and the main transport protein for Zn, was also adjusted for (HR 1.79; 95% CI: 1.15, 2.78; *P*-trend = 0.009). No associations were observed with the Cu/Se-ratio (Table 2).

**Table 2:** Risk of pneumonia death according to the tertiles of serum Cu/Zn- and Cu/Se-ratio and Cu, Zn and Se concentration

Serum parameter	Tertile of serum parameter			*P*-trend
	1	2	3	
**Serum Cu/Zn-ratio**	0.48–1.08	1.09–1.27	1.28–2.97	
N of events/subjects	33/697 (4.7%)	46/696 (6.6%)	60/695 (8.6%)	
IR/1000 PY	2.03	2.92	4.17	
Model 1	1	1.38 (0.88, 2.15)^a^	2.06 (1.35, 3.15)	0.001
Model 2	1	1.30 (0.83, 2.03)	1.75 (1.13, 2.71)	0.010
**Serum Cu/Se-ratio**	3.94–9.37	9.38–11.90	11.91–31.82	
N of events/subjects	35/696 (5.0%)	51/694 (7.3%)	53/698 (7.6%)	
IR/1000 PY	2.17	3.34	3.53	
Model 1	1	1.66 (1.06, 2.58)	1.80 (1.06, 3.01)	0.058
Model 2	1	1.47 (0.94, 2.31)	1.49 (0.87, 2.55)	0.235
**Serum Cu (µmol/L)**	7.24–15.89	15.90-18.25	18.26–36.51	
N of events/subjects	38/685 (5.5%)	41/720 (5.7%)	60/683 (8.8%)	
IR/1000 PY	2.38	2.52	4.24	
Model 1	1	1.07 (0.69, 1.67)	1.88 (1.25, 2.82)	0.002
Model 2	1	1.03 (0.66, 1.61)	1.64 (1.08, 2.50)	0.016
**Serum Zn (µmol/L)**	8.26–13.61	13.62–15.14	15.15–22.94	
N of events/subjects	64/653 (9.8%)	36/761 (4.7%)	39/674 (5.8%)	
IR/1000 PY	4.62	2.09	2.55	
Model 1	1	0.44 (0.29, 0.66)	0.60 (0.40, 0.89)	0.010
Model 2	1	0.50 (0.33, 0.75)	0.66 (0.44, 0.99)	0.038
**Serum Se (µmol/L)**	0.46–1.22	1.23–1.47	1.48–3.05	
N of events/subjects	58/695 (8.3%)	32/710 (4.5%)	49/683 (7.2%)	
IR/1000 PY	3.74	2.06	3.19	
Model 1	1	0.56 (0.33, 0.95)	0,81 (0.45, 1.45)	0.462
Model 2	1	0.56 (0.33, 0.96)	0.88 (0.49, 1.58)	0.653

^a^ Values are hazard ratio (95% confidence interval)

IR, incidence rate; PY, person-years

Model 1: adjusted for age and examination year

Model 2: adjusted for model 1 and history of coronary heart disease, stroke, diabetes, liver or pancreas disease (yes/no); smoking (never smoker, previous smoker, current smoker < 20 cigarettes/day, current smoker *≥* 20 cigarettes/day); education (years); income (euros/year); intake of alcohol (g/week); leisure-time physical activity (kcal/day); and body mass index

In addition, we investigated the associations of the risk of pneumonia death with serum concentrations of Cu, Zn and Se separately. After adjustment for potential confounders, the multivariate HR in the highest compared with the lowest tertile of serum Cu was 1.64 (95% CI: 1.08, 2.50; *P*-trend = 0.016) (Table 2, model 2). With serum Zn concentrations, the lowest risk was observed in the second tertile, without further decrease in risk in the highest tertile (Table 2). Serum Se concentration was not associated with risk of pneumonia mortality (Table 2).

In the sensitivity analyses after excluding men with history of ischemic heart disease, stroke, cancer, diabetes, or liver or pancreas disease (n of remaining men = 1514, 89 deaths due to pneumonia) serum Cu concentration had, after adjustment for potential confounders, a stronger association with pneumonia death than in the main analyses. The multivariate-adjusted HR in the highest vs. the lowest serum Cu tertile was 2.45 (95% CI: 1.41, 4.23; *P*-trend < 0.001) (Supplementary Table 3, model 2). Additionally, Cu/Zn-ratio was associated with a slightly higher risk of pneumonia death than in the main analyses. The multivariate-adjusted HR in the highest compared to the lowest Cu/Zn-ratio was 1.91 (95% CI: 1.10, 3.32; *P*-trend = 0.017) (Supplementary Table 3, model 2). However, contrary to the main analysis, serum Zn concentration did not show a statistically significant association. Consistent with the main analyses, there was no statistically significant association between the Cu/Se-ratio and serum Se concentration with the risk of pneumonia death (Supplementary Table 3, Model 2).

## Discussion

In this prospective, population-based cohort study, we found that a higher serum Cu concentration and a higher Cu/Zn-ratio were associated with increased pneumonia mortality in middle-aged and older men in Eastern Finland. Conversely, we observed an inverse relationship between serum Zn concentration and pneumonia death, with a statistically significant trend across tertiles. However, we observed a threshold effect, with the lowest risk of pneumonia death in the second tertile of serum Zn concentration and no additional risk reduction in the highest tertile. On the other hand, we observed no statistically significant association between the serum Se concentration or Cu/Se-ratio and the risk of pneumonia death.

It has been suggested that high serum Cu concentrations may adversely affect respiratory health [16]. Possibly, the prooxidant effect of Cu may contribute to our finding that high serum Cu concentrations are associated with an increased risk of pneumonia-related mortality. Our results are consistent with previous findings that increased serum Cu concentrations may be a predictive marker of an increased risk of incident infections [15] or pneumonia [7]. The average serum Cu concentration observed in this study (17.4 µmol/L) is consistent with the average serum Cu concentrations reported for men in multiple European countries [17].

Zn has been shown to mitigate inflammation in pneumonia [18], with serum Zn concentration above 10.7 µmol/L being linked to a decreased pneumonia incidence [19]. In this study we observed a statistically significant inverse association between serum Zn concentration and pneumonia death. In our study population, only 36 men (1.7%) had serum Zn concentrations below 10.7 µmol/L, indicating Zn deficiency [20]. In general, the mean serum Zn concentration at baseline of our study, 14.4 µmol/L, corresponds well with the normal range found in other studies of elderly subjects [20–22]. Our results are consistent with the previous finding that a normal serum Zn concentration > 10.7 µmol/L at baseline is associated with a lower risk of all-cause mortality in the elderly [19]. Similarly, a reduction of mortality risk was observed with elderly study subjects supplemented with high dose of Zn [22].

In the present study, the high serum Cu/Zn-ratio is explained by increased serum Cu concentration. Zn and Cu and their relation play pivotal roles in defending the body from oxidative stress [23]. Different pathologies have a greater effect on serum concentrations of Cu and Zn than nutrition, except in cases of overt deficiency or supplementation [8]. Indeed, Cu/Zn-ratio is considered a marker of oxidative stress, inflammation and aging [6] as well as one of the most sensitive biological markers of several pathologies [8]. It has been proposed that serum Cu/Zn-ratio would be a more sensitive marker in various pathologies than serum Cu and Zn concentrations individually [24]. Consistent with our results, a high serum Cu/Zn-ratio has been proposed as a predictive marker for incident infections [15], pneumonia [7], and disease severity and its prognosis in COVID-19 patients [9].

A deficiency of Se has been linked to impaired innate and adapted immunity and increased infection susceptibility [25]. Due to the inverse regulation of serum Cu and Se concentrations under inflammation, the Cu/Se-ratio has been proposed as a more sensitive biomarker than the individual concentrations of serum Cu or Se [26]. However, the existing literature of the Cu/Se-ratio as a novel biomarker remains limited. It has been reported as a potential biomarker for ageing [27], neonatal infections [28], and a range of inflammatory conditions [26, 29]. In our study, neither the Cu/Se ratio nor serum Se concentration were statistically significant predictors of pneumonia-related death. This observation may be explained by the fact that 99.6% (*n* = 2072) of our study subjects had serum Se concentrations of 0.6 µmol/L or higher, which is the lower limit of the reference value in Finland for normal plasma Se [30]. A systematic review of nine experimental human studies has proposed that plasma Se concentrations around 1.3 µmol/L can be considered beneficial, while higher concentrations may be associated with null or adverse effects [31]. The mean serum Se concentration in this study was 1.3 µmol/L. The adequate serum Se concentrations observed in our study population may be attributed at least in part to Finland’s Se supplementation in fertilizers since 1984. This supplementation has led to a significant increase in the population’s mean plasma Se concentration [32].

### Strengths and limitations

This study has several strengths, including a population-based approach, prospectively collected data, extensive examinations for potential confounders, and a long follow-up with an adequate number of pneumonia deaths. The potential weakness is the single measurement of serum Cu, Zn and Se concentrations at baseline, which may have changed over the long follow-up period. This would induce random error and attenuate the associations. As the study population consisted only of middle-aged and older men from Eastern Finland, caution should be exercised with generalization of the results. This is especially true for sex, age, and different ethnicities. As a result of the large variations in Se levels in soil globally, there is also a wide variation in Se status in different populations, with many having suboptimal serum Se concentrations [33]. Serum Se concentration is considered a biomarker of short-term Se status, although under reasonably stable conditions it can also serve as a measure of long-term Se intake [34]. Although serum Zn concentration is not an ideal biological marker of Zn status, it is considered the best existing biomarker of Zn deficiency in the population [35]. In addition, serum metal concentrations differ between the sexes. Women have significantly higher Cu/Zn-ratio [36] and Cu concentration presumably due to hormonal influence, while Zn concentration is higher in men [37, 38]. Furthermore, pneumonia incidence is higher in men, but varies widely between and even within countries and increases with advancing age [27].

## Conclusions

In conclusion, our findings suggest that increased serum Cu/Zn-ratio and increased serum Cu concentration may predict higher risk of pneumonia death in middle-aged and older men in Eastern Finland. Conversely, serum Zn concentration showed an inverse relationship, suggesting a potential protective effect against pneumonia death. In contrast, serum Se concentrations and Cu/Se-ratios were not associated with pneumonia death. Our research design is observational and therefore cannot infer causality. Further studies in diverse populations and ideally with repeated measurements of serum Cu, Zn and Se over the course of follow-up are necessary to validate our findings and further evaluate the clinical relevance of Cu/Zn-ratio and serum Cu and Zn concentration as predictive markers of pneumonia death and to possibly integrate the measurement of serum Cu and Zn as a standard measurement in the risk groups.

## Electronic supplementary material

Below is the link to the electronic supplementary material.

Supplementary Material 1

## Data Availability

The data used in this study will not be openly available because it contains sensitive personal information of the participants that cannot be completely anonymized. However, the analytical code used for the current study can be made available upon reasonable request.

## Abbreviations

BMI: Body mass index
CI: Confidence interval
CRP: C-reactive protein
Cu: Copper
Cu/Se-ratio: Copper to selenium ratio
Cu/Zn-ratio: Copper to zinc ratio
CVD: Cardiovascular disease
HR: Hazard ratio
IR: Incidence rate
KIHD: Kuopio Ischaemic Heart Disease Risk Factor Study
PY: Person-years
SD: Standard deviation
Se: Selenium
Zn: Zinc
