# Supplementary material for: Serum copper, zinc and selenium and their ratios as predictors of pneumonia death risk in men: the Kuopio Ischaemic Heart Disease Risk Factor Study
Source: Infection. 2025 Jul 10;53(6):2557–64. doi: 10.1007/s15010-025-02596-8 (PMC12675712; doi:10.1007/s15010-025-02596-8)
Supplement: Supplementary file 1 — Supplementary Material 1 [file 15010_2025_2596_MOESM1_ESM.docx]

Supplementary Information

**Serum copper, zinc and selenium and their ratios as predictors of pneumonia death risk in men: The Kuopio Ischaemic Heart Disease Risk Factor Study**

Jaakko T Laine, Tomi-Pekka Tuomainen, Jukka T Salonen, Jyrki K Virtanen

Correspondence to Jyrki K. Virtanen. University of Eastern Finland, Institute of Public Health and Clinical Nutrition, P.O. Box 1627, 70211 Kuopio, Finland. Tel. +358 40 355 2957, E-mail: jyrki.virtanen@uef.fi.

| **Supplementary Table 1** Baseline characteristics of 2088 KIHD participants by tertiles of serum Cu/Zn-ratio. | | | | |
| --- | --- | --- | --- | --- |
|  | Serum Cu/Zn-ratio tertile | | |  |
| Characteristic | 1 (0.48–1.08) | 2 (1.09–1.27) | 3 (1.28–2.97) |  |
| Participants, *n* | 697 | 696 | 695 |  |
| Sociodemographic and lifestyle factors |  |  |  |  |
| Age, years | 52.1 (5.4)^b^ | 52.9 (5.1) | 53.5 (4.9) |  |
| Body mass index, kg/m^2^ | 26.8 (3.4) | 27 (3.5) | 26.8 (3.6) |  |
| Leisure time physical activity, kcal/day | 148 (172) | 142 (164) | 131 (172) |  |
| Education, years | 9.2 (3.8) | 8.6 (3.4) | 8.4 (3.2) |  |
| Income, Euros/year | 14 605 (9833) | 13 536 (8607) | 12 318 (8349) |  |
| Alcohol intake, g/week | 57 (101) | 62 (89) | 100 (149) |  |
| Current smoker, % | 21 | 26 | 40 |  |
| Multivitamin use, % | 2.0 | 0.4 | 0.7 |  |
| Disease history and serum biomarkers |  |  |  |  |
| History of ischemic heart disease, stroke, diabetes, liver or pancreas disease % | 24 | 30 | 31 |  |
| Serum C-reactive protein, mg/L | 1.2 (1.2) | 1.7 (1.6) | 2.5 (2.1) |  |
| Serum albumin, g/L | 43.1 (3.4) | 42.4 (3.5) | 41.6 (3.5) |  |
| Dietary intakes |  |  |  |  |
| Energy intake, kcal/day | 2445 (589) | 2454 (602) | 2410 (647) |  |
| Meat g/d^c^ | 158 (85) | 158 (80) | 160 (77) |  |
| Dairy g/d | 709 (357) | 714 (359) | 704 (353) |  |
| Fish g/d | 41 (48) | 45 (53) | 52 (61) |  |
| Eggs, g/d | 34 (26) | 32 (25) | 31 (26) |  |
| Grains, g/d | 261 (92) | 258 (92) | 243 (95) |  |
| Vegetable margarines and oils, g/d | 22 (18) | 20 (18) | 17 (15) |  |
| Butter, g/d | 30 (25) | 33 (26) | 35 (28) |  |
| Fruits, berries, vegetables, roots, g/d | 436 (180) | 421 (173) | 398 (182) |  |
| Zinc, mg/d^d^ | 15 (3) | 15 (3) | 15 (3) |  |
| ^a^*P*-trend was calculated by using linear regression (continuous variables) or by chi-square test (categorical variables). | | | | |
| ^b^Values are means (SD) or percentages. | | | | |
| ^c^Includes red meat, white meat, game and offal. | | | | |
| ^d^Values adjusted for total energy intake using the residual method. | | | | |

| **Supplementary Table 2** Baseline characteristics of 2088 of KIHD participants by tertiles of serum Cu/Se-ratio. | | | | |
| --- | --- | --- | --- | --- |
|  | Serum Cu/Se-ratio tertile | | |  |
| Characteristic | 1 (3.94–9.37) | 2 (9.38–11.90) | 3 (11.91–31.82) |  |
| Participants, *n* | 696 | 694 | 698 |  |
| Sociodemographic and lifestyle factors |  |  |  |  |
| Age, years | 51.5 (6.3)^b^ | 52.6 (5.4) | 54.3 (2.7) |  |
| Body mass index, kg/m^2^ | 26.5 (3.2) | 27.1 (3.6) | 26.9 (3.8) |  |
| Leisure time physical activity, kcal/day | 150 (169) | 147 (170) | 126 (169) |  |
| Education, years | 9.4 (3.7) | 8.8 (3.4) | 8.0 (3.3) |  |
| Income, Euros/year | 15 702 (9934) | 13 589 (9057) | 11 176 (7219) |  |
| Alcohol intake, g/week | 67 (109) | 81 (134) | 71 (106) |  |
| Current smoker, % | 21 | 29 | 36.7 |  |
| Multivitamin use, % | 2.4 | 0.6 | 0.1 |  |
| Disease history and serum biomarkers |  |  |  |  |
| History of ischemic heart disease, stroke, diabetes, liver or pancreas disease % | 23.1 | 27.5 | 33.7 |  |
| Serum C-reactive protein, mg/L | 1.2 (1.1) | 2.0 (1.9) | 2.2 (2.0) |  |
| Serum albumin, g/L | 43.0 (3.7) | 42.0 (3.4) | 42.1 (3.4) |  |
| Dietary intakes |  |  |  |  |
| Energy intake, kcal/day | 2443 (591) | 2451 (639) | 2415 (608) |  |
| Meat g/d^c^ | 165 (86) | 164 (80) | 147 (75) |  |
| Dairy g/d | 669 (356) | 715 (350) | 743 (360) |  |
| Fish g/d | 42 (50) | 50 (56) | 46 (56) |  |
| Eggs, g/d | 30 (22) | 34 (28) | 34 (26) |  |
| Grains, g/d | 264 (98) | 253 (93) | 245 (87) |  |
| Vegetable margarines and oils, g/d | 23 (18) | 20 (17) | 17 (15) |  |
| Butter, g/d | 27 (24) | 33 (27) | 38 (27) |  |
| Fruits, berries, vegetables, roots, g/d | 428 (178) | 417 (177) | 409 (181) |  |
| Zinc, mg/d^d^ | 16 (4) | 15 (3) | 15 (3) |  |
| ^a^*P*-trend was calculated by using linear regression (continuous variables) or by chi-square test (categorical variables). | | | | |
| ^b^Values are means (SD) or percentages. | | | | |
| ^c^Includes red meat, white meat, game and offal. | | | | |
| ^d^Values adjusted for total energy intake using the residual method. | | | | |

| **Supplementary Table 3** Risk of pneumonia death according to the tertiles of serum Cu/Zn- and Cu/Se-ratio and Cu, Zn and Se concentration after exclusion of the men with history of coronary heart disease, stroke, diabetes, liver or pancreas disease at baseline | | | | |
| --- | --- | --- | --- | --- |
|  | **Tertile of serum parameter** | | |  |
| **Serum parameter** | 1 | 2 | 3 | *P*-trend |
| **Serum Cu/Zn-ratio** | 0.48-1.08 | 1.09-1.27 | 1.28-2.97 |  |
| N of events/subjects | 20/504 (4.0%) | 29/505 (5.7%) | 40/505 (7.9%) |  |
| IR/1000 PY | 1.65 | 2.43 | 3.60 |  |
| Model 1 | 1 | 1.40 (0.79-2.48)^a^ | 2.15 (1.25-3.68) | 0.004 |
| Model 2 | 1 | 1.31 (0.74-2.32) | 1.91 (1.10-3.32) | 0.017 |
|  | | | | |
| **Serum Cu/Se-ratio** | 3.94-9.37 | 9.38-11.90 | 11.91-31.82 |  |
| N of events/subjects | 23/504 (4.6%) | 29/505 (5.7%) | 37/505 (7.3%) |  |
| IR/1000 PY | 1.93 | 2.49 | 3.21 |  |
| Model 1 | 1 | 1.34 (0.76-2.34) | 1.74 (0.90-3.35) | 0.105 |
| Model 2 | 1 | 1.19 (0.67-2.10) | 1.52 (0.78-2.94) | 0.213 |
|  | | | | |
| **Serum Cu (μmol/L)** | 7.87-15.74 | 15.75-18.10 | 18.11-30.06 |  |
| N of events/subjects | 19/496 (3.8%) | 26/512 (5.1%) | 44/506 (8.7%) |  |
| IR/1000 PY | 1.58 | 2.17 | 3.93 |  |
| Model 1 | 1 | 1.39 (0.77-2.51) | 2.68 (1.57-4.60) | <0.001 |
| Model 2 | 1 | 1.31 (0.72-2.37) | 2.45 (1.41-4.23) | <0.001 |
|  | | | | |
| **Serum Zn** **(μmol/L)** | 8.26-13.61 | 13.62-15.14 | 15.15-21.57 |  |
| N of events/subjects | 41/532 (7.7%) | 23/494 (4.7%) | 25/488 (5.1%) |  |
| IR/1000 PY | 3.44 | 1.96 | 2.16 |  |
| Model 1 | 1 | 0.57 (0.34-0.95) | 0.73 (0.44-1.20) | 0.169 |
| Model 2 | 1 | 0.63 (0.38-1.06) | 0.77 (0.46-1.29) | 0.287 |
|  | | | | |
| **Serum Se** **(μmol/L)** | 0.56-1.23 | 1.24-1.47 | 1.48-3.05 |  |
| N of events/subjects | 34/501 (6.8%) | 21/494 (4.3%) | 34/519 (6.6%) |  |
| IR/1000 PY | 2.88 | 1.87 | 2.80 |  |
| Model 1 | 1 | 0.90 (0.47-1.75) | 1.48 (0.72-3.04) | 0.299 |
| Model 2 | 1 | 0.88 (0.45-1.71) | 1.58 (0.77-3.23) | 0.285 |
| ^a^ Values are hazard ratio (95% confidence interval). | | | | |
| IR, incidence rate; PY, person-years. | | | | |
| Model 1: adjusted for age and examination year. | | | | |
| Model 2: adjusted for model 1 and smoking (never smoker, previous smoker, current smoker <20 cigarettes/day, current smoker >20 cigarettes/day); education (years); income (euros/year); intake of alcohol (g/week); leisure-time physical activity (kcal/day); and body mass index. | | | | |

Eligible participants for sensitive analyses, n=1514

Participants excluded with history of CHD, stroke, liver or pancreas disease, or diabetes (n=574)

Participants excluded (n=594)

- Missing serum Cu, Zn or Se concentrations n=128
- Serum CRP>10 mg/L (n=71)
- Chronic bronchitis (n=193)
- Lung tuberculosis (n=88)
- Bronchial asthma (n=60)
- History of cancer (n=39)
- Rheumatoid arthritis (n=15)

Eligible participants for analyses between serum Cu, Zn, Se concentrations and pneumonia death, n=2088

The whole study population,

n=2682

**Supplemental Figure 1**

Selection of participants for the current analyses among men aged 42–60 y in 1984–1989 from the Kuopio Ischaemic Heart Disease Risk Factor Study
